# Transcriptomic responses of bat cells to European bat lyssavirus 1 infection under conditions simulating euthermia and hibernation

**DOI:** 10.1186/s12865-023-00542-7

**Published:** 2023-04-21

**Authors:** Markéta Harazim, Juliette Perrot, Hugo Varet, Hervé Bourhy, Julien Lannoy, Jiri Pikula, Veronika Seidlová, Laurent Dacheux, Natália Martínková

**Affiliations:** 1 Institute of Vertebrate Biology, Czech Academy of Sciences, Květná 8, 60300 Brno, Czechia; 2 Department of Botany and Zoology, Masaryk University, Kotlářská 2, 61137 Brno, Czechia; 3 Institut Pasteur, Université Paris Cité Lyssavirus, Epidemiology and Neuropathology Unit, 28 rue du Docteur Roux, 75724 Paris Cedex 15, France; 4 Institut Pasteur, Université Paris Cité Bioinformatics and Biostatistics Hub, 28 rue du Docteur Roux, 75724 Paris Cedex 15, France; 5 Department of Ecology and Diseases of Zoo Animals, Game, Fish and Bees, University of Veterinary Sciences Brno, Palackého třída 1946/1, 61242 Brno, Czechia; 6 RECETOX, Masaryk University, Kotlářská 2, 61137 Brno, Czechia

**Keywords:** Chiroptera, *Myotis myotis*, Hibernation, Lyssaviruses, In vitro infection model, EBLV-1, Innate immunity, Heat shock proteins (HSPs), Antiviral state, Transcriptome

## Abstract

**Background:**

Coevolution between pathogens and their hosts decreases host morbidity and mortality. Bats host and can tolerate viruses which can be lethal to other vertebrate orders, including humans. Bat adaptations to infection include localized immune response, early pathogen sensing, high interferon expression without pathogen stimulation, and regulated inflammatory response. The immune reaction is costly, and bats suppress high-cost metabolism during torpor. In the temperate zone, bats hibernate in winter, utilizing a specific behavioural adaptation to survive detrimental environmental conditions and lack of energy resources. Hibernation torpor involves major physiological changes that pose an additional challenge to bat-pathogen coexistence. Here, we compared bat cellular reaction to viral challenge under conditions simulating hibernation, evaluating the changes between torpor and euthermia.

**Results:**

We infected the olfactory nerve-derived cell culture of *Myotis myotis* with an endemic bat pathogen, European bat lyssavirus 1 (EBLV-1). After infection, the bat cells were cultivated at two different temperatures, 37 °C and 5 °C, to examine the cell response during conditions simulating euthermia and torpor, respectively. The mRNA isolated from the cells was sequenced and analysed for differential gene expression attributable to the temperature and/or infection treatment. In conditions simulating euthermia, infected bat cells produce an excess signalling by multitude of pathways involved in apoptosis and immune regulation influencing proliferation of regulatory cell types which can, in synergy with other produced cytokines, contribute to viral tolerance. We found no up- or down-regulated genes expressed in infected cells cultivated at conditions simulating torpor compared to non-infected cells cultivated under the same conditions. When studying the reaction of uninfected cells to the temperature treatment, bat cells show an increased production of heat shock proteins (HSPs) with chaperone activity, improving the bat’s ability to repair molecular structures damaged due to the stress related to the temperature change.

**Conclusions:**

The lack of bat cell reaction to infection in conditions simulating hibernation may contribute to the virus tolerance or persistence in bats. Together with the cell damage repair mechanisms induced in response to hibernation, the immune regulation may promote bats’ ability to act as reservoirs of zoonotic viruses such as lyssaviruses.

## Background

Immune response requires both energy and resources, and becomes prohibitively costly in hibernation. Hibernating animals in torpor reduce their body temperature, heart and respiratory rate, lower their metabolism, and modify their immune response to survive fasting in the winter months spent in hibernation [1–5]. At the cellular level, avoiding infection overload as well as energy depletion in torpor requires precise regulation of cellular and humoral inflammatory responses to the presence of pathogens [6–9]. Hibernators reduce the number of circulating leukocytes in torpor [1], but increase their number in mucosal tissues [10], promoting early sensing and elimination of pathogens upon their invasion, and avoiding a costly systemic response.

The metabolism/immune response trade-off at the molecular level evolves in favour of suppressed inflammation and infection tolerance [11–13]. Although expression of pro-inflammatory cytokines increases during torpor [10], their levels fluctuate during cyclic arousals in conjunction with increased expression of anti-inflammatory cytokines [14].

Bats represent a specific group of hibernators that has modified pathogen sensing through adaptive evolution of pattern recognition receptors (PRR). The PRR adaptation enables early pathogen invasion recognition and functional adaptations in immune regulation. Following pathogen sensing, bats modify the cytokine production to avoid a strong pro-inflammatory response [15]. Interferon regulatory factors directly influence inflammation following viral infection [12, 16] and the interferon (IFN) type I expression is unusually high without viral stimulation, in contrast to other mammals [17] (but see [18]). Adaptations in innate interferon regulators have been shown to diminish the immune response to viruses in bats [19]. These adaptations can be found in functional consensus at the level of immune genes expression regulation [17, 20], enabling bats to maintain immune homeostasis to thrive in changing environmental conditions.

Bats may be asymptomatic after lyssavirus infection, including rabies virus (RABV) and European bat lyssavirus 1 (EBLV-1), and unlike other mammals, they might survive the natural exposure to lyssaviruses, demonstrated by the production of post-infection antibodies. As a result, lyssavirus antibody seroprevalence can be surprisingly high in populations of reservoir bats [21–23], and the titer of rabies antibodies can increase with each re-infection [24]. Bats also produce lyssavirus neutralizing antibodies after exposure to aerosolised RABV virus and survive the infection, while in some cases, mice develop rabies [25].

The most widespread and represented bat lyssavirus in Europe remains EBLV-1, and *Eptesicus serotinus* is considered the main reservoir [26]. Other bat species, including *Myotis myotis*, can be infected or exposed to the EBLV-1 infection [22]. Natural EBLV-1 infection in mammals other than bats through spill-over events remains limited to a few examples that include sheep [27], cats [28] and a stone marten (*Martes foina*) [29]. Only two confirmed human cases of rabies induced by EBLV-1 were described [30].

Experimental infection study conducted in mice shows that EBLV-1 infection induced more severe inflammatory changes in the brain, similar to infection with European bat lyssavirus 2 (EBLV-2), but different to RABV [31]. In addition, the CCL2, CCL5 and CXCL10 chemokine patterns in the brain were variable in the respective lyssavirus infections, with a greater expression of CCL5 in vivo and CXCL10 production in vitro with EBLV infection, whereas CCL2 and CCL5 proteins were more abundant in vitro with RABV infection [32]. Only one study conducted on the common pipistrelle investigated the influence of hibernation on EBLV-1 infection, and demonstrated an influence in the duration of the incubation period and the distribution of viruses in extraneural tissues [33].

Bat survival after lyssavirus infection appears to vary according to the host species, but also intraspecifically. For example, rabid *Eptesicus* bats naturally infected with EBLV-1 are routinely diagnosed, demonstrating that they can be susceptible to infection and die of rabies [26, 34], but survival cases are also reported [35]. Circulation of EBLV-1 in *M. myotis* colonies is not correlated with greater morbidity and mortality [22, 36]. This observation suggests that bats use different mechanisms, including physiological and behavioral, to cope with the virus infection. The ability of certain bats to survive the EBLV-1 infection, or the ability of EBLV-1 persistence in bat colonies, could be associated with specific epidemiological factors [22, 35], virus strain differences in terms of pathogenesis [37, 38], and specific bat immunological response.

Despite the strong focus on the topic, understanding the physiological reaction to lyssavirus infection in mammals, and more especially in bats, is limited due to an endangered status of many species and the lack of a suitable *in vivo* model. Since systemic response to infection in torpor is suppressed during evolution [11, 39], we were interested in the onset of antiviral state in infected bat cells in conditions corresponding to euthermia and hibernation. To study the reaction of bat cells to lyssavirus infection, we designed an experiment simulating the effect of lyssavirus infection on a bat cell line cultivated at different conditions. The olfactory nerve derived cell lines of *M. myotis*, MmNOl [40], served as a model system to study infection by the EBLV-1, a lyssavirus present in *M. myotis* populations across Europe [21, 22, 41].

We hypothesize that bat cells will mount an innate immune response to lyssavirus infection, dependent on cultivation conditions. When cultivated at conditions simulating euthermia, the molecular response of infected bat cells will be qualitatively and quantitatively different from the response in cells cultivated at low temperatures, simulating torpor, where a regulatory response will occur.

## Results

### RNA sequencing and data processing

**Fig. 1:**
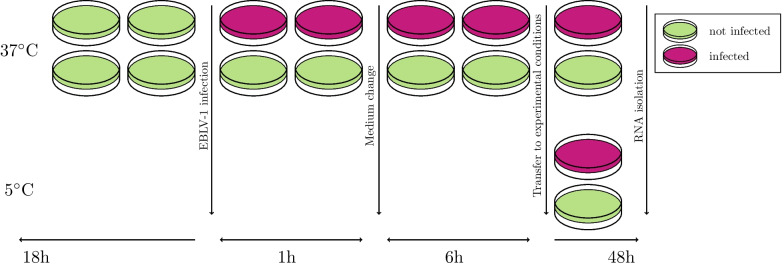
Experimental design of cultivation of bat cells at temperatures simulating euthermia and torpor. Green—untreated cell culture, magenta—cell culture infected with EBLV-1

We purified total RNA from the infected and control samples cultivated at both euthermia and hibernation simulating conditions at 54 hpi (6 h + 48 h, see Fig. 1). We obtained reads from 12 samples treated with different conditions (Fig. 1). We mapped the reads on the *Myotis lucifugus* genome (Ensembl:Myoluc2.0) obtaining 24,859 gene counts of 292,980,660 mapped reads, with an average of 982.15 reads per gene.

### Control of virus infection over experimental conditions

**Fig. 2:**
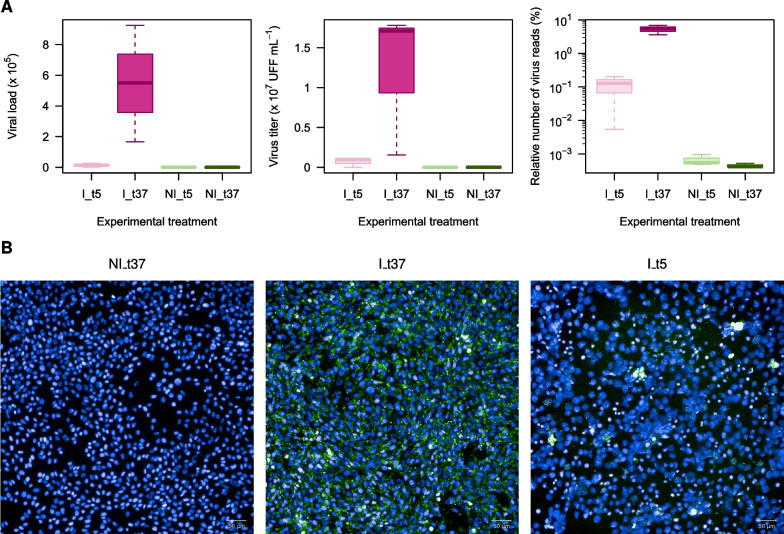
EBLV-1 infection in the experimental treatments of the *Myotis myotis* olfactory nerve cells MmNOl. **A** Measured viral load in 500 ng of RNA, virus titer in supernatant and proportion of reads mapped to EBLV-1 reference. Note that virus RNA and virus production is present after 48 h cultivation at 5 $^\circ$C, demonstrating that the cells at 5 $^\circ$C were infected. **B** Fluorescent images of the cell culture (blue) infected with the EBLV-1 virus (green). I – infected, NI – not infected, t – cultivation temperature

For each experimental condition and replicate, the presence of EBLV-1 (strain 8918FRA) RNA in infected cells was controlled by the quantification of the viral load by specific qPCR targeting the viral nucleoprotein (N) mRNA (Table 1) and by the percentage of reads mapping to the genome sequence of the 8918FRA strain. Both results were consistent, demonstrating a high level of infection and replication for the cells infected and incubated at 37 $^\circ$C, with an average of $5.5 \times 10^5$ (min: $1.7 \times 10^5$, max: $9.2\times 10^5$) N-mRNA copies for 500 ng of total RNA and around 5.3% (mean $1.8 \times 10^6$) of virus reads (Fig. 2). The presence of viral RNA in infected cells incubated 48 h at 5 $^\circ$C was observed at a lower level and associated with more variability, with a mean value of $1.4 \times 10^4$ (min: $0.08\times 10^4$, max: $26 \times 10^4$) N-mRNA copies for 500 ng of total RNA and on average 0.1% (mean value $3.9\times 10^4$) of virus reads (Fig. 2). No viral RNA was detected by qPCR in all non-infected cells incubated either at 37 $^\circ$C, or at 5 $^\circ$C, and the percentages of mapped reads were all below 0.001%, corresponding to background signal.

**Table 1:** Primers used for RT-qPCR verification

Gene	Forward	Reverse
*HSPA5*	AAGACAATCATCTCCTGGGAAC	TGCCATTCACGTCTATCTCAAA
*HSPA8*	GCCCAAGGTCCAAGTAGAAT	GTAACAGTCTTCCCAAGGTAGG
*HSPA9*	AAGAGAGACGGGAGTTGATTTG	CACAGATGAGGAGAGTTCACAC
*HSPB8*	GGTAAAGACCAAGGACAGATACG	CCGCAGGAAGCTGGATTT
*HSPD1*	CTACTGGTGGTGCTGTGTTT	TCTTTGGTCACAATGACCTCTC
*HSPG2*	GTCACTGAGGCTCCAAGTAAG	TGGCTGTGCAGATGAAAGT
*HSPH1*	GGATGAGAAACCTCGGATAGTG	GCTGTTCCCAGTACCTTCAA
*8918FRA_N*	CCCTGCCATCAAAGACAAGA	AGCATTCATCCCTGACAAGATAG

The presence of virus infection was also confirmed by the detection of the presence of the N protein by direct immunofluorescence in the infected cells (Fig. 2). The number of infected cells was higher (nearly 100%) for the cells incubated at 37 $^\circ$C compared to the cells placed at 5 $^\circ$C, consistent with the N-mRNA quantification. Absence of N protein was observed in non-infected cells, either at 37 $^\circ$C (Fig. 2) or 5 $^\circ$C.

Lastly, we checked the presence of infectious viruses released in the supernatant of the infected cells by virus titration. At 37 $^\circ$C, the mean virus titer was high, with $1.2 \times 10^7$ FFU mL$^{-1}$ (min: $1.6\times 10^6$, max: $1.8\times 10^7$), compared to the 5 $^\circ$C condition, for which the mean virus titer was $6.9\times 10^6$ FFU mL$^{-1}$, with higher variability (min: $6.3\times 10^3$, max: $1.1\times 10^6$) (Fig. 2). Non-infected cells did not contain any infectious virus particles.

### Differential expression and pathway analysis

**Fig. 3:**
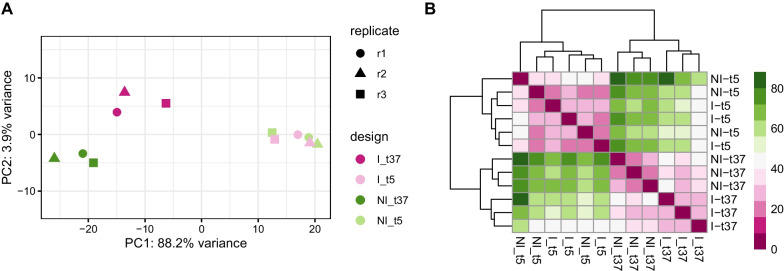
**A** Principal components of the gene expression profiles obtained from the sequencing data. **B** Sample to sample dissimilarity of the sequencing data inferred from complete-linkage clustering based on Euclidean distances. I – infected, NI – not infected, t – cultivation temperature

Exploration of the overall effects in the expression data using the principal component analysis (PCA) showed that the temperature treatment was responsible for the highest biological variation in gene expression. The PCA of the 12 samples corrected for batch effect showed 88.2% of variation along the PC1 axis, separating the samples based on temperature treatment and 3.9% variation along the PC2 axis, separating the samples based on infection treatment (Fig. 3).

#### Reaction of MmNOl cells to EBLV-1 infection in euthermia

In cells cultivated at 5 $^\circ$C, we found no significant difference in the gene expression between the infected and noninfected cells ($\textit{p} > 0.05$; cf. Fig. 3).

**Fig. 4:**
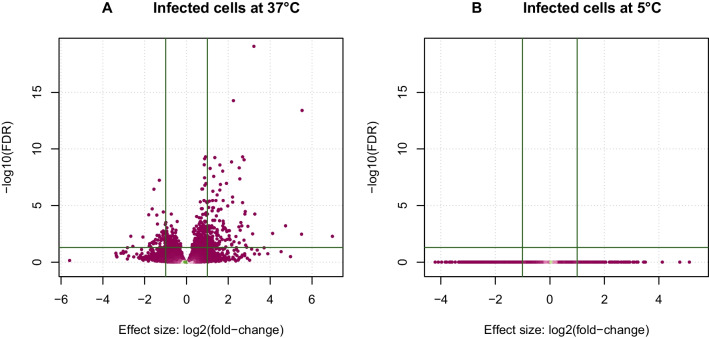
Differential expression between the infection-treated and control cells cultivated **A** at 37 $^\circ$C and **B** at 5 $^\circ$C. Horizontal lines – $\text{FDR}=0.05$, vertical lines – $\log _2(FC)>1$

In contrast, we found 200 genes upregulated genes in the MmNOl cells after infection by EBLV-1 cultivated at 37 $^\circ$C (Fig. 4, Additional file 1). The upregulated genes were overrepresented in 14 pathways ($\textit{p}\le 0.05$; Table 2, Additional file 2). The highest $\log _2(FC)>3$ was recorded in *NLRP10* encoding NOD-like receptor family, pyrin domain containing 10, *TP63* encoding tumor protein p63, *MFAP5* encoding microfibril associated protein 5, *STMN4* encoding stathmin 4, *BCAS1* encoding breast carcinoma-amplified sequence 1, *GABBR2* encoding gamma-aminobutyric acid type B receptor subunit 2 and *INHBE* encoding inhibin subunit beta E.

We identified 90 downregulated genes (Additional file 1) in the infected samples cultivated at 37 $^\circ$C compared with the noninfected control samples cultivated at the same conditions. The highest $-\log _2(FC)>2$ was observed in *SNED1* encoding sushi, nidogen and EGF-like domains 1, *GP1BB* (glycoprotein Ib), and *CPNE5* encoding calcium-dependent membrane-binding protein.

#### Transcriptomic reaction to simulated hibernation in MmNOl cells

We identified 2,957 genes differentially expressed between the temperature-treated non-infected samples. We identified 1,713 downregulated genes in the non-infected samples cultivated at 5 $^\circ$C compared to the non-infected controls cultivated at 37 $^\circ$C (Additional file 4). The downregulated gene set did not show any over-representation in any functional pathways. The genes with the highest $-\log _2(FC)$ included a high number (17) of HOX gene paralogs.

The 1,244 upregulated genes in the cold-treated samples (Additional file 4) were significantly overrepresented in 25 functional pathways ($\text{FDR}\le 0.05$; Table 3). The upregulated genes included a number of heat-shock proteins (*HSPB7, HSPG2, HSPA9, HSPD1, HSPH1, HSPA5, HSPA8*) and a HSP-binding co-chaperone *HSPB8*.

#### Transcriptome of EBLV-1 infection in simulated hibernation of MmNOl cells

Finally, we evaluated the effect of infection in the samples cultivated at 5 $^\circ$C compared to samples cultivated at 37 $^\circ$C corrected for temperature effects (i.e. infected samples (I-t5 vs I-t37) vs control samples (NI-t5 vs NI-t37)). We identified 36 upregulated genes, most notably with $\log _2(FC) > 2$ for *BAIAP3* encoding BAI1-associated protein 3, and *TXNIP* encoding thioredoxin interacting protein. Furthermore, upregulated expression was detected in *KIT*, *CXCL12*, *MAP2K6*, *PIK3IP1*, *WIPI1*, *YPEL3*, *TNFRSF17*, *THEMIS2* and *ATP1B2* (full list in Additional file 3).

The downregulated genes with the highest changes in expression levels with $-\log _2(FC) > 3$ included *STMN4*, *RASGRP3* encoding ras guanyl-releasing protein 3, *GABBR2* and *BCAS1*. Other downregulated genes (full list in Additional file 4) included *TNFSF18*, *IL15RA* and *ATP1B2*. The 157 downregulated genes in the infected cells irrespective of the temperature effect were significantly overrepresented in 4 pathways in the 5 $^\circ$C cultivated cells (Table 4, Additional file 5).

### Quantitative PCR

We validated the results from the RNAseq experiments with RT-qPCR on a set of selected genes (Table 1, Additional file 6). The panel included six members of the heat shock protein family, with *HSPA5*, *HSPA8* and *HSPA9* (members 5, 8 and 9 of the family A – Hsp70), *HSPB8* (member 8 of the family B – small), *HSPD1* (member 1 of the family D – Hsp60) and *HSPH1* (member 1 of the family H – Hsp110), the gene *HSPG2* (heparan sulfate proteoglycan 2) and *GABBR2* (Gamma-aminobutyric acid type B receptor subunit 2). The RT-qPCR results correspond to the RNAseq results (Additional file 6).

## Discussion

The circulation of a virulent and pathogenic agent in natural wildlife populations is constrained by the limited survival of the host and the host ability to suppress the pathogen multiplication. Bats are a likely natural host to lyssaviruses and show species-specific responses to the natural infection [42, 43] that might facilitate pathogen persistence in host populations. As the natural reservoir, they have undergone coevolution affecting both the host and the pathogen, resulting in lower pathogenicity, but higher replication rates of bat lyssavirus strains compared to other hosts [44]. Epidemic cycles of the EBLV-1 infection in bats do not influence temporal changes in *M. myotis* [22] or *Eptesicus serotinus* survival [35], but experimental injections of *Eptesicus* species with EBLV-1 occasionally lead to lethal infections [37, 45].

Being a long-lived group with low reproduction rate and many endangered species, bats do not represent a practical model species. The sources of information on physiology, immunity and molecular reaction to lyssavirus infection in bats are consequently limited. The *in vivo* experimental infection of *Eptesicus spp.* bats were used to study viral transmission via different infection routes (e. g. intramuscular and subcutaneous) by the EBLV-1 resulting in immune response (seroconversion) in some, but not all, individuals [37, 45]. The immortalized bat derived cell lines can be used as an *in vitro* model with limited biological variation, enabling the analysis of the impact of different incubation conditions on the cell reaction. The study of reaction to infection in bats can thus be approximated by using bat-derived cell cultures [40], which can be infected in a controlled environment using a temperature treatment simulating hibernation torpor and euthermia.

EBLV-1 infections have been described in multiple bat species, including *M. myotis* [21, 41, 46], where repeated EBLV-1 infections were recorded within populations [22]. Based on naturally occurring infections of bats by lyssaviruses and their prevalence, we selected *in vitro* model of EBLV-1 infection, *M. myotis*, derived cells to infection by EBLV-1 in simulated hibernation and euthermia. We found that the expression profiles of bat cells reflected the cultivation temperature more than the EBLV-1 infection status. Results of the experiments with cells cultivated at 37 $^\circ$C formed a distinct and divergent cluster on PC1, separated from the results of cells cultivated at 5 $^\circ$C (Fig. 3). The infected cells had expression profiles more similar between the temperature treatments than the non-infected controls.

### Response to temperature treatment

Adaptations are required for bats to face the temperature extremes associated with being actively flying mammals in ecozones with a large seasonal temperature differential [2, 47]. Bats respond to the changing external conditions in their life span by adopting mechanisms allowing their cells to survive the rapid changes of organismal milieu. Our results show that bat cells subjected to low cultivation temperatures upregulated the production of heat-shock proteins, contributing to cellular health and survival by maintaining molecular stability and function. HSPs are essential for bat survival in high temperatures during flight [48], and seem to have an equally important role in the low temperatures of simulated hibernation.

Cultivating the MmNOl bat cells in hibernation-like conditions at low temperatures showed upregulation in a number of rRNA-processing pathways (Table 3). The rRNA-processing genes were also reported to be upregulated in hibernating dwarf lemurs [49]. The high expression of these genes can be related to ribosomal damage, which would lead to a need for replenishment of functional ribosomes.

We also detected a strong role of nonsense-mediated mRNA decay in the simulated hibernation. As the cells face the stress of low temperatures, transcription can be impacted with the need to control for the missense transcripts [50]. The structural misfolding can occur in proteins as well, which is controlled by the chaperone activity of the upregulated heat shock proteins [51].

### Response to EBLV-1 infection

The infection of the cells at the euthermic state induced a strong expression of genes involved in several antiviral response pathways affecting the cell cycle, immune response, and apoptosis. The TGF-$\beta$ pathway, overrepresented in the upregulated gene set, induces anti-inflammatory cytokine production in macrophages that are phagocytosing apoptotic cells [52], and its activity is supplemented by the upregulated SMAD2/SMAD3:SMAD4 pathway [53]. TGF-$\beta$ and related pathways were previously shown to be crucial in differentiation of the immune response to the attenuated strains from pathogenic rabies viruses. While the pathogenic virus in mice does not affect the TGF-$\beta$ response, the attenuated virus infection leads to upregulation of TGF-$\beta$ expression and to immune activation in mice [54]. The upregulation of TGF-$\beta$ in the bat cell line infected by pathogenic EBLV-1 might have a similar effect to reaction induced by the immunization using attenuated virus vaccines in other mammals. The activation of the related pathways may lead the immune response to keeping the viral load low and enabling the coexistence of the bat hosts and EBLV-1.

*TCF7* (encoding T-cell factor 1), a gene upregulated in the infected tissues, controls survival of Treg cells and functions as an immune regulator [55]. The Treg/Th17 cell subsets are more prevalent in bats compared to mice or humans [56], suggesting a role of the Treg/Th17 balance regulation in the differing bat immune response. The TGF-$\beta$ pathway in the neural tissue may perform a similar function of negative immune regulators on the resident cells with immune activity, such as glial cells.

Regulation of Wnt signalling by *RUNX3*, which our analysis suggested occurring in cells infected by EBLV-1, can supplement the role of the above-mentioned immune regulators, as it promotes Treg survival. However, Wnt regulation can also be linked to other pathways, where it affects insulin sensitivity. Related insulin-like growth factor binding proteins (IGFBPs) signalling system promotes the survival of neurons [57].

The immune reaction to EBLV-1 infection is supplemented by upregulated activity in collagen tissues and extracellular matrix structures. Extracellular matrix undergoes remodelling during infection to ensure the availability of receptors and intercellular contacts [58]. The pathways related to collagen and the extracellular matrix can thus serve as a physiological mechanism of defence from infection in non-immune cells and in healing.

The differential expression analysis between the infected samples cultivated at both conditions and stripped of the effect of temperature showed several upregulated genes reacting to the infection during the simulated hibernation. *BAIAP3* and *YPEL3* are both p53 target genes with a role in cell cycle and apoptosis regulation. *TXNIP* serves as an oxidative stress regulator, which can react to the stress related to the changing temperatures. The immune activity in the cells infected and subjected to simulated hibernation seems to be suppressed.

Apoptosis of infected cells could also be enhanced in hibernation by downregulation of *IL15RA*. The upregulated chemokine receptor *KIT* and chemotactic *CXCL12* promotes macrophage differentiation. The synergy of these mechanisms can cause apoptosis in infected cells as a mechanism to lower the infection spread, which is followed by macrophage-mediated phagocytosis of apoptotic cells and anti-inflammatory signalling of TGF-$\beta$. This possibly prevents the rapid release of pro-inflammatory cytokines, leading to tolerance of the cells to viral infections during hibernation. Similar results of adaptation to viral infection by tolerance without the induction of the adaptive immune response has been recently observed in *Rousettus aegyptiacus* in response to their naturally harboured Marburg virus [59].

The lack of reaction in the infected cells cultivated at 5 $^\circ$C may implicate low virus load caused by inhibition of replication and infection of brain tissue in the low body temperatures during hibernation. Following low virus replication during torpor, bats that are infected during hibernation may be capable of quick virus clearance upon arousal. This mechanism would suggest that hibernating bats are acquiring resistance to clinical rabies via inoculation during hibernation. Low infection doses of lyssaviruses in bats do not cause infection symptomatology, but they can lead to seropositivity [60].

## Conclusions

The changes in gene expression under the condition of simulated hibernation in the cell culture showed adaptations of the repair mechanisms of structural changes caused by the cold. These adaptations may enable the cells to survive in changing temperature conditions without the need of the organism to expedite more energy to renew the cells. The transcriptome of bat cells infected by EBLV-1 suggests a tolerative anti-inflammatory response to infection. Together with the mechanism of adaptations developed in response to other pathogens endemic to bats, such as positive selection in multiple immune genes [61–63], this can lead to bat tolerance of the virus presence. Apoptotic pathways can ensure the early clearance and limited replication of the virus. The anti-inflammatory reaction of macrophages that clear out the apoptotic cells can reduce the cost of mounting adaptive immunity. Keeping the immune system balanced, and the virus load low, may contribute to the coexistence of circulating lyssaviruses and bats.

## Methods

### Experimental inoculations and RNA purification

The experiments were carried out using a previously established *M. myotis* cell line [40] derived from the olfactory nerve (MmNOl cells). The bat cell cultures were cultivated in $\hbox {Gibco}$ DMEM, High Glucose, $\hbox {GlutaMAX}$ medium (Thermo Fisher Scientific) with 10% fetal bovine serum (FBS) supplement (Thermo Fisher Scientific) at 37 $^\circ$C with 5% CO$_2$. The cell-adapted EBLV-1 strain 8918FRA [64] was used, and virus stock was prepared on BSR cells.

The cells were cultivated in two groups; at 37 $^\circ$C, to simulate euthermia and at 5 $^\circ$C to simulate torpor. Each of the temperature groups contained an infected sample and a non-infected control (Fig. 1). The experimental design thus consisted of cells under four experimental conditions. All of them were inoculated in independent laboratory session triplicates to prevent technical errors in the results.

An amount of $2.5 \times 10^6$ cells was seeded on T25 flask. At 18 h after seeding, the cells were infected with lyssavirus EBLV-1 8918FRA at multiplicity of infection (MOI) 1 (i.e., $2.5 \times 10^6$ fluorescent focus-forming unit (FFU) in 700 $\mu$L of medium without FBS. After 1 h, the cell culture medium was replaced with a fresh, virus-free medium containing 10% FBS and the cells were incubated for 6 h at 37 $^\circ$C with 5% CO$_2$. Then, the cells were incubated 48 h at 37 $^\circ$C with 5% CO$_2$ or at 5 $^\circ$C after closing the flasks.

### RNA extraction and quantification

After a total of 54 h post-infection (hpi), the supernatants were collected and stored at −80 $^\circ$C. The cells were lysed with 1 mL of TRIzol (ThermoFisher) and RNA was extracted using Direct-zol RNA Miniprep Kit (Zymo Research) according to the manufacturer’s instructions (including a DNAse I treatment), and eluted into 50 $\mu$L RNAse free water. The concentration of RNA was measured by photometric quantification using Nanodrop (Version 2000/2000c, Thermo Scientific) and the RNA integrity was evaluated using 2100 Bioanalyzer system (Agilent) which provides an RNA integrity number. Strand-specific single-end cDNA libraries were prepared according to the manufacturers’ instructions (TruSeq Stranded mRNA sample prep kit, Illumina) and sequenced using Illumina NextSeq 500 sequencer.

### Virus titration

The supernatants (20 $\mu$L) collected from the cells cultured under the different conditions were successively 1:5 diluted in DMEM 10% FBS medium (80 $\mu$L) in a 96-well plate. A total of 50,000 BSR cells (50 $\mu$L) was then dispensed into each well, and incubated for 48 h at 37 $^\circ$C under 5% CO$_2$. Then, the supernatant was removed and the cells were fixed with 80% acetone for 30 min at 4 $^\circ$C. After a short air drying step, the cells were immunolabelled after incubation for 30 min at 37 $^\circ$C with 25 $\mu$L polyclonal FITC-conjugated antibody directed against the rabies virus nucleoprotein (BioRad, Cat. No. 3572112) at twice the concentration recommended by the manufacturer. The cells were then washed three times with PBS and the fluorescence foci were counted under UV microscope. The virus titration was based on technical duplicates and expressed in FFU per mL.

### Viral load quantification

For each condition of cell cultures, the 8918FRA viral load were determined by a specific N-EBLV-1 qPCR using an absolute quantification method. A quantity of 500 ng (11 $\mu$L) of purified RNA extracted from each experiment was converted into complementary DNA (cDNA), using the Superscript III reverse transcriptase (Invitrogen) according to the manufacturer’s instructions. For this step, RNA was first incubated at 65 $^\circ$C for 5 min with 1 $\mu$L of 10 mM dNTP mix (Invitrogen) and 1 $\mu$L of 50 $\mu$M of oligo(dT)12-18 (Invitrogen), then placed on ice. The complementary step was performed with the addition of 1 $\mu$L of Superscript III Reverse transcriptase (Invitrogen), 4 $\mu$L of 5$\times$ First-Strand Buffer, 1 $\mu$L of 0.1 M DTT and 1 $\mu$L of RNAsin ribonuclease inhibitor (Promega) for a final volume of 20 $\mu$L. The mix was incubated at 50 $^\circ$C for 60 min and the reaction was inactivated by heating at 70 $^\circ$C for 15 min. The qPCR was performed using 2 $\mu$L of cDNA or diluted plasmids and a mix containing 5 $\mu$L of Power SYBR Green PCR Master Mix (Applied Biosystems), 0.88 $\mu$L of each 8918FRA_N primer (Table 1) and nuclease free water for a final volume of 10 $\mu$L. Technical triplicates were amplified using a QuantStudio 6 Flex System thermocycler with the following conditions: initial denaturation step (1$\times$ repetition, 10 min, 95 $^\circ$C), amplification step (40$\times$ repetitions, 15 sec 95 $^\circ$C, 1 min 60 $^\circ$C) and melting curve determination step (1$\times$ repetition, 15 sec, 95 $^\circ$C, 1 min 60 $^\circ$C, 15 sec 95 $^\circ$C, 15 sec 60 $^\circ$C). The determination of viral load (copy number of N mRNA) was performed using a standard curve generated after 1:10 successive dilution of a plasmid containing the N gene of the 8918FRA strain (from 1.10^6^ to $1.10^{-2}$ copies, in triplicate).

**Table 2:** Upregulated pathways in the EBLV-1 infected MmNOl cells cultivated at 37 $^\circ$C

Pathway name	$p_\text{FDR}$
IGF-2 mRNA Binding Proteins (IGF2BPs/IMPs/VICKZs) bind RNA	5.91E-05
Extracellular matrix organization	5.91E-05
Response of EIF2AK1 (HRI) to heme deficiency	1.41E-04
Elastic fibre formation	2.10E-03
Signaling by TGFB family members	2.62E-03
RUNX3 regulates WNT signaling	2.62E-03
Crosslinking of collagen fibrils	4.81E-03
Collagen formation	5.31E-03
Assembly of collagen fibrils and other multimeric structures	9.70E-03
SMAD2/SMAD3:SMAD4 heterotrimer regulates transcription	2.85E-02
Integrin cell surface interactions	3.00E-02
Glycoprotein hormones	3.00E-02
Downregulation of ERBB4 signaling	3.50E-02
Transcriptional regulation by RUNX3	3.50E-02

### Immunofluorescence

A total of $3.5\times 10^4$ cells well$^{-1}$ was seeded into 96-well-plates (655086, Greiner Bio) in 200 $\mu$L of DMEM medium supplemented with 10% FBS. After 24 h at 37 $^\circ$C under 5% CO$_2$, the cells were infected with the 8918FRA strain at MOI 1 in 50 $\mu$L of DMEM medium without FBS. Then, the protocol was similar to the one described with the T25 flask, with 1 h of incubation at 37 $^\circ$C under 5% CO$_2$, following by another incubation of 6 h after replacing the medium with 200 $\mu$L of DMEM supplemented with 10 FBS, and lastly 48 h incubation either at 37 $^\circ$C under 5% CO$_2$ or at 5 $^\circ$C. After this last incubation, cells were fixed using 4% paraformaldehyde fixative solution (J61984, Alfa Aesar) for 20 min at 5 $^\circ$C. Cells were washed three times with PBS and immunolabelled after incubation for 30 min at 37 $^\circ$C with 25 $\mu$L of a polyclonal FITC-conjugated antibody directed against the rabies virus nucleoprotein (BioRad, Cat. No. 3572112) at twice the concentration recommended by the manufacturer. The cells were then washed three times with PBS and the fluorescence foci were counted under UV microscope. The virus titration was based on technical duplicates and expressed in fluorescence focus-forming unit per mL (FFU mL$^{-1}$). Cells were stained for 15 min with Hoechst (1:10000) (H1399, Thermo Scientific). After another step of three PBS washes, images were obtained from Opera Phenix (PerkinElmer) with 20$\times$ air objective lens and analyzed in Columbus Image Data Management System (PerkinElmer).

**Table 3:** Upregulated pathways in the MmNOl cells cultivated at 5 $^\circ$C compared to the 37 $^\circ$C cultivated controls

Pathway name	$p_\text{FDR}$
rRNA processing in the nucleus and cytosol	1.63E-13
Major pathway of rRNA processing in the nucleolus and cytosol	2.17E-13
rRNA processing	2.17E-13
rRNA modification in the nucleus and cytosol	1.69E-10
Response of EIF2AK4 (GCN2) to amino acid deficiency	1.95E-06
Translation	3.39E-06
Metabolism of RNA	6.24E-04
Cellular response to starvation	1.22E-03
SRP-dependent cotranslational protein targeting to membrane	3.55E-03
GTP hydrolysis and joining of the 60S ribosomal subunit	3.55E-03
L13a-mediated translational silencing of Ceruloplasmin expression	3.55E-03
Nonsense Mediated Decay (NMD) independent of the EJC	3.55E-03
Eukaryotic Translation Elongation	3.81E-03
Peptide chain elongation	4.08E-03
Eukaryotic Translation Initiation	4.08E-03
Cap-dependent Translation Initiation	4.08E-03
Formation of a pool of free 40S subunits	5.04E-03
Eukaryotic Translation Termination	5.04E-03
Collagen formation	9.17E-03
Selenocysteine synthesis	1.05E-02
Syndecan interactions	1.06E-02
Response of EIF2AK1 (HRI) to heme deficiency	1.06E-02
Mitochondrial translation elongation	1.13E-02
Mitochondrial translation termination	1.13E-02
Collagen biosynthesis and modifying enzymes	1.26E-02
Mitochondrial translation	1.26E-02
Nonsense-Mediated Decay (NMD)	1.44E-02
Nonsense Mediated Decay (NMD) enhanced by the EJC	1.44E-02
PERK regulates gene expression	1.51E-02
IGF-2 mRNA Binding Proteins (IGF2BPs/IMPs/VICKZs) bind RNA	1.56E-02
Viral mRNA Translation	2.07E-02
SMAD2/SMAD3:SMAD4 heterotrimer regulates transcription	2.35E-02
Mitochondrial translation initiation	2.60E-02
Non-integrin membrane-ECM interactions	4.67E-02

### Quantification of the virus reads

The fastq files corresponding of each experimental condition triplicate were used to determine the number and the percentage of total virus reads after a cleaning step and the mapping of cleaned reads on the genome sequence of EBLV-1 8918FRA strain (GenBank: EU293112) performed with CLC Assembly Cell (Qiagen, Hilden, Germany), in a dedicated workflow built on the Galaxy platform of Institut Pasteur [65] and adapted to single-read format [66].

**Table 4:** Downregulated pathways in the EBLV-1 infected MmNOl cells cultivated at 5 $^\circ$C, corrected for temperature effects

Pathway name	$p_\text{FDR}$
Response of EIF2AK1 (HRI) to heme deficiency	8.18E-05
IGF-2 mRNA Binding Proteins (IGF2BPs/IMPs/VICKZs) bind RNA	2.67E-04
Extracellular matrix organization	2.52E-02
Crosslinking of collagen fibrils	4.11E-02

### RNA sequencing and data processing

The sequencing library preparation and sequencing were performed as a service by the Institut Pasteur Core Facility. The raw sequencing data was processed using the RNA-seq pipeline from Sequana [67]. Sequencing reads were cleaned of adapter and low-quality sequences using cutadapt version 2.6 [68]. STAR version 2.7.3a [69] was used for the alignment onto the reference genome (*Myotis lucifugus* 2.0 on Ensembl 99, Myoluc2.0). The featureCounts version 2.0.0 [70] was used to count the number of reads mapped on each gene.

### Differential expression and pathway analysis

We analysed the differential expression of the RNA gene counts among the temperature and infection treated samples using R package DESeq2 [71]. The read counts were normalized around the median, with normalization scaling factors in the range of 0.74$-$1.36. Negative binomial distribution generalised linear models were fitted in DESeq2 to obtain the model coefficients that can be interpreted as $\log _2(FC)$ (fold change).

Independent filtering was used to retrieve the results, which were then adjusted for multiple testing using Benjamini-Hochberg false discovery rate method (FDR). Genes that were significantly differentially expressed at $\textit{p}\le 0.05$ (after the FDR correction) and $\log _2(FC)\ge 1$ were used for downstream analyses.

We analysed the pathways involved in the bat cell line response to infection by EBLV-1 using manually curated Reactome databases and pathway analysis tool [72]. We evaluated the pathways significantly overrepresented ($\textit{p}\le 0.05$) in the dataset in our further investigation.

### Quantitative PCR

A list of 8 differentially expressed genes was selected, based on the RNAseq analysis. cDNA synthesis and quantitative PCR based on the detection of the SYBR Green dye was performed as previously described with the viral load quantification. All the samples were measured in triplicates. Gene expression levels were normalized to the endogenous expression of the geometric mean of two housekeeping genes, *EEF1A1* and *PGK1*. The $\Delta$Ct was calculated by subtracting the geometric mean of the above-mentioned housekeeping genes from the Ct values of the selected genes [73]. Variations in gene expression were calculated as the *n*-fold change in expression in the samples depending of the experimental conditions (e. g. infected versus non-infected at 37 $^\circ$C) using the $2^{- \Delta \Delta Ct}$ method [74]. The $\Delta \Delta$Ct was calculated by normalizing the $\Delta$Ct values to the corresponding reference samples.

## Supplementary information

Additional file 1. Differential gene expression in infected cells cultivated at 37 °C compared to noninfected controls cultivated at 37 °C.

Additional file 2. Pathways overrepresented in the upregulated genes in infected cells cultivated at 37 °C compared to noninfected controls cultivated at 37 °C.

Additional file 3. Differential gene expression in infected cells cultivated at 5 °C, corrected for temperature effect.

Additional file 4. Differential gene expression in noninfected cells cultivated at 5 °C compared to cells cultivated at 37 °C.

Additional file 5. Pathways overrepresented in the upregulated genes in noninfected cells cultivated at 5 °C compared to noninfected cells cultivated at 37 °C.

Additional file 6. Differential gene expression confirmation by RT-qPCR in cells under different treatments.

## Data Availability

The cDNA reads generated and analysed during the study are available in the NCBI GEO repository accession number GSE228404.

## Abbreviations

Ct: Cycle threshold
DMEM: Dulbecco’s modified eagle medium
EBLV-1: European bat lyssavirus 1
FBS: Fetal bovine serum
FC: Fold change
FDR: False discovery rate
FFU: Fluorescent-focus units
FITC: Fluorescein isothiocyanate
hpi: Hours post-infection
HSP: Heat-shock protein
I-t5: Infected cells cultivated at 5 $^\circ$C
I-t37: Infected cells cultivated at 37 $^\circ$C
MOI: Multiplicity of infection
N-tI5: Non-infected cells cultivated at 5 $^\circ$C
NI-t37: Non-infected cells cultivated at 37 $^\circ$C
PC: Principal component
PCA: Principal component analysis
PRR: Pattern-recognition receptor
RABV: Rabies virus
RT-qPCR: Reverse transcription quantitative polymerase chain reaction
UV: Ultra-violet
