# Supplementary material for: Transcriptomic responses of bat cells to European bat lyssavirus 1 infection under conditions simulating euthermia and hibernation
Source: BMC Immunol. 2023 Apr 21;24:7. doi: 10.1186/s12865-023-00542-7 (PMC10120247; doi:10.1186/s12865-023-00542-7)
Supplement: Supplementary file 6 — Additional file 6. Differential gene expression confirmation by RT-qPCR in cells under different treatments. [file 12865_2023_542_MOESM6_ESM.pdf]

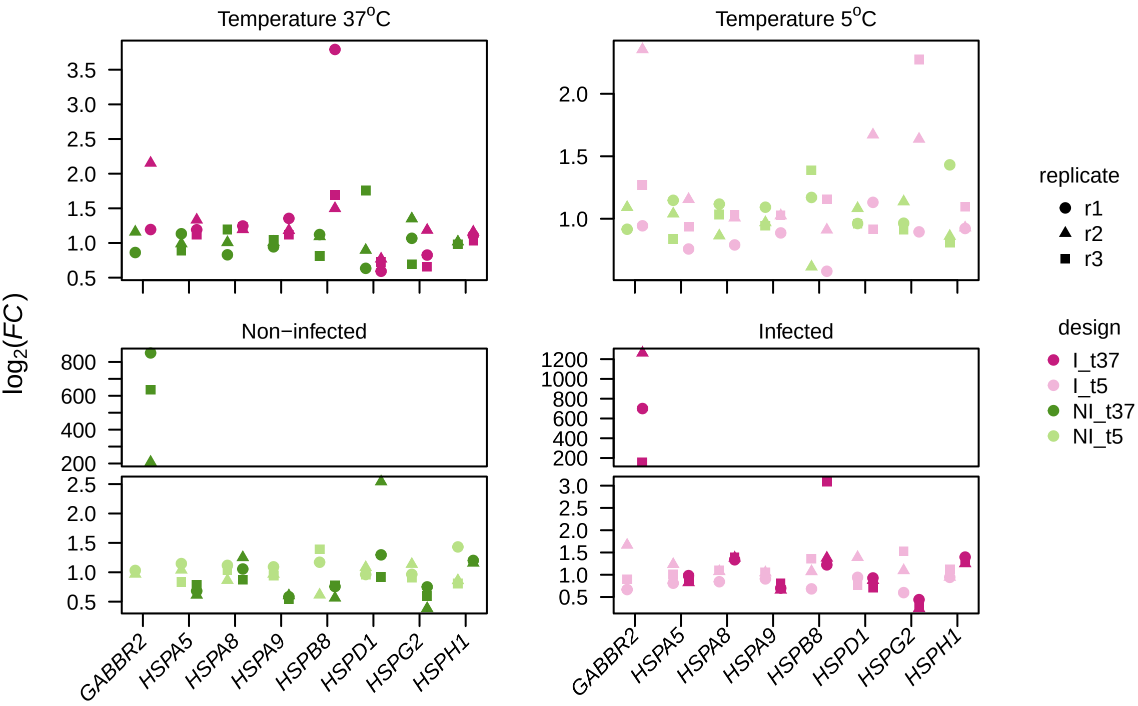

Temperature 37°C
Temperature 5°C
Non−infected
Infected
log2(FC)
replicate
r1
r2
r3
design
I_t37
I_t5
NI_t37
NI_t5
GABBR2
HSPA5
HSPA8
HSPA9
HSPB8
HSPD1
HSPG2
HSPH1
